# Validation of the 24-item recovery assessment scale-revised (RAS-R) in the Norwegian language and context: a multi-centre study

**DOI:** 10.1186/s12955-018-0849-3

**Published:** 2018-01-25

**Authors:** Eva Biringer, Marit Tjoflåt

**Affiliations:** 1 Department of Research and Innovation, Helse Fonna Local Health Authority, PO Box 2170, N-5504 Haugesund, Norway; 2 Folgefonn Community Mental Health Centre, Division of Psychiatry, Helse Fonna Local Health Authority, Sjukehusvn. 14, N-5451 Valen, Norway

**Keywords:** Recovery, Mental health, Psychometric, Validation, Instrument, Assessment, Evaluation

## Abstract

**Background:**

The Recovery Assessment Scale-revised (RAS-R) is a self-report instrument measuring mental health recovery. The purpose of the present study was to translate and adapt the RAS-R into the Norwegian language and to investigate its psychometric properties in terms of factor structure, convergent and discriminant validity and reliability in the Norwegian context.

**Methods:**

The present study is a cross-sectional multi-centre study. After a pilot test, the Norwegian version of the RAS-R was distributed to 231 service users in mental health specialist and community services. The factor structure of the instrument was investigated by a confirmatory factor analysis (CFA), and internal consistency was assessed by Cronbach’s alpha.

**Results:**

The RAS-R was found to be acceptable and feasible for service users. The original five-factor structure was confirmed. All model fit indices, including the standardised root mean square residual (SRMR), which is independent of the χ^2^-test, met the criteria for an acceptable model fit. Internal consistencies within sub-scales as measured by Cronbach’s alpha ranged from 0.65 to 0.85. Cronbach’s alpha for the total scale was 0.90. As expected, some redundancy between factors existed (in particular among the factors Personal confidence and hope, Goal and success orientation and Not dominated by symptoms).

**Conclusions:**

The Norwegian RAS-R showed acceptable psychometric properties in terms of convergent validity and reliability, and fit indices from the CFA confirmed the original factor structure. We recommend the Norwegian RAS-R as a tool in service users’ and health professionals’ collaborative work towards the service users’ recovery goals and as an outcome measure in larger evaluations.

## Background

Earlier research has shown little correlation between personal recovery, as defined by service users, and the staff-rated tools frequently used as outcome measures in mental health [1, 2]. Andresen and colleagues showed that recovery measures are measuring a unique construct that is not comprehensively assessed by conventional clinical measures [1]. Recovery in mental health can be defined as ‘a way of living a satisfying, hopeful and contributing life even with limitations caused by illness’ [3]. It is conceptualised as both a process and an outcome, and improvement is not only reflected in changes in the state of the disorder (resolution) but can also be seen as an adjustment of life to work around the disorder (readjustment) or an adaptation to living with the disorder (redefinition) [4]. Hope and optimism about the future, purpose and meaningful activity, positive identity, connectedness and empowerment are central features of recovery [5–8]. However, no current ‘gold-standard’ measure of recovery exists [9, 10], and existing measures of recovery vary with regard to the constructs covered and their psychometric properties [9–13].

The Recovery Assessment Scale (RAS) is a self-report instrument measuring personal recovery that was developed more than 20 years ago by Giffort and colleagues in the US [14, 15]. At present, it is one of the most widely used measures of personal recovery [10–12, 16, 17]. Giffort and colleagues combined participatory action research and narrative analysis to generate a 41-item scale with adequate test–retest reliability and internal consistency [14, 15]. A study involving 1824 persons with serious mental health illness concluded that 24 of these items represented a meaningful five-factor solution [18]. The RAS-R thus consists of 24 items on five-level scales (‘Strongly Disagree’, ‘Disagree’, ‘Not Sure’, ‘Agree’, ‘Strongly Agree’) [18]. These items can be added up to produce summary scales representing five dimensions of personal recovery: Personal confidence and hope (items 7, 8, 9, 10, 11, 12, 13, 14 & 21), Willingness to ask for help (items 18, 19 & 20), Goal and success orientation (items 1, 2, 3, 4 & 5), Reliance on others (items 6, 22, 23 & 24), Not dominated by symptoms (items 15, 16 & 17) and a total scale. Sub- and total summary scales are frequently converted to mean scale scores [13].

Several systematic reviews have recommended the RAS as a tool for assessing personal recovery [11, 12], including as a routine tool in clinical settings [11]. A user-informed review performed by Law and colleagues (2012) concluded that the RAS is the most acceptable and valid measure currently available [9]. In their review of studies reporting psychometric properties of the RAS, Salzer and Brusilovskiy (2014) concluded that means and standard deviations across 28 studies were fairly consistent [13]. In longitudinal investigations included in their review, the instrument was found to be sensitive to change over time, and results for internal consistency of the scale, test–retest and inter-rater reliabilities were very good. Further, the factor structures found were consistent across studies. Based on these results, they recommended the instrument as a measure of recovery in clinical evaluations and research. There is still need for recovery research in the Nordic countries [19]. However, to conduct such research, instruments measuring personal recovery should be available in Nordic languages. The purpose of the present study was therefore to translate and adapt the RAS-R for use in a Norwegian context and to investigate its psychometric properties in terms of factor structure, convergent and discriminant validity and reliability.

## Methods

### Translation and adaptation of the RAS-R to the Norwegian language and context

Translation of the original RAS-R version into the Norwegian language was performed by an authorised translation bureau. Minor adaptations and adjustments of wordings were done after two service users and five health professionals commented on the first translated version. Lingual aspects, cultural adaption and cognitive issues were discussed by the project team, and the resulting version was then piloted with the help of nine service users at a day care unit in a community mental health centre. Five of the service users were women and four were men, their mean age was 30 years (range 19–50) and they all had severe mental health conditions (psychosis spectrum disorders). They completed the instrument with the co-author MT as observer.

The participants in the pilot study experienced the questions as easily comprehensible. Several participants indicated that they felt the questions were particularly relevant for them, and completing the instrument obviously provoked reflections about the participants’ personal recovery stories. However, some minor issues related to the comprehensibility and acceptability of some items were revealed among the participants. Like this participant, some found the interpretation of item 2, ‘I have my own plan for how to stay or become well’, to be challenging:

*‘Plan? No... I have small plans throughout the day, but it's like overall plan you mean? I'm trying to understand what you mean … I'll answer unsure as to whether I have a plan, at least I don’t have a big plan.’*

Most comments mentioned item 5, ‘I have a purpose in life’. In this context ‘purpose’ could be translated into Norwegian as ‘mål’ (‘goal’), ‘formål’ (purpose), ‘hensikt’ (‘intention’) and/or ‘mening’ (‘meaning’). For instance, a young man who wished for a better social life and someone special to share his life with, found delimiting the item conceptually difficult:

*‘…. I'm not sure, not about the question, but it’s a bit “big” what they are asking about, almost as if they are asking about the meaning of life …’*

After discussing the feedback from the participants in the pilot study, the project team choose the term ‘formål’, which translates into ‘goal’, ‘meaning’ and ‘purpose’, as this term was perceived as the closest to the meaning of the item included in the original U.S. English version.

Item 11 (‘I have an idea of who I want to become’) was also confusing for some:

*‘I have an idea of what I want ... Don't I want to become myself, then? [] But if it is education ..., [or] is it as a person ..? I think it’s a bit of a vague question ...’*

Item 18 (‘I know when to ask for help’), item 19 (‘I am willing to ask for help’) and item 20 (‘I ask for help when I need it’) were perceived as overlapping by some participants. Finally, two participants found the negative sentences in item 6 (‘Even when I don’t care about myself, other people do’), item 7 (‘Fear doesn’t stop me from living the way I want to’) and item 15 (‘Coping with my mental illness is no longer the main focus of my life’) a bit confusing.

Based on feedback from the pilot study and discussions among the members of the project team, minor changes to wordings were made to the translated version in order to make the items clearer and more grammatically sound. A back-translated version was evaluated by the research team, and a few minor alterations in the introductory text were made, but no further changes were made to the items. The resulting Norwegian version of the RAS-R is included as Additional file 1.

### Study design and setting

To investigate the psychometric properties of the translated version of the RAS-R, we conducted a cross-sectional multi-centre study in the mental health specialist and community services in the Haugaland and Sunnhordland regions on the west coast of Norway. The respondents were recruited from one municipality, four community mental health centres (CMHCs, i.e. secondary services) and one psychiatric hospital (i.e. tertiary service) according to predefined criteria (Table 1). The participating institutions varied in size and were situated in both urban and rural areas. As personal recovery is a longitudinal process [4, 20], we aimed at including participants representative of all stages of the recovery process. Therefore, a sub-sample of service users (*n* = 85) who had been using mental health services 2 years ago, and who presumably had experienced partial or total recovery, were invited to participate. Data were collected from Spring 2015 until Autumn 2016.

**Table 1:** Overview of mental health care units, inclusion criteria, participation rate and way of completing the RAS-R

Unit	Inclusion criteria	*n* (%) screened	*n* (%) included	Way of completing RAS-R
Mental health hospital with co-located CMHC for adults	Severe mental health condition and contact with mental health services > 6–12 months	135 (42)	112 (35)	Pencil and paper
Two co-organised CMHCs for adults		66 (21)	52 (16)	
Mental health services in one municipality	Severe mental health condition	36 (11)	32 (10)	
CMHC for adults	Used mental health services 2 years ago due to any mental health condition needing specialist care	85 (26)	35 (11)	Telephone interview
SUM		322 (100)	231 (72)	

*CMHC* Community Mental Health Centre

### Participants

The survey aimed to include ten respondents for each of the 24 items of the RAS-R. In all, 322 potential participants were regarded by their therapists as relevant to include according to the inclusion criteria. Of these, 231 (72%) agreed to participate and provide informed consent. The participants received services at specialist level or had regular contact with community mental health services. The mental health services, inclusion criteria, participation rate and method of completing the RAS-R are shown in Table 1. The participants were asked to complete the 24-item RAS-R questionnaire along with information about their age, gender, level of education, civil status and employment status. In addition, the participants were asked to respond to the general question ‘In your experience, where in your process of recovery are you now, compared to the situation when things were at the worst (=1), and how you wish that your situation should ideally be (=10)?’ Their responses were recorded on a visual analogue scale ranging from 1 to 10. As there is no currently available gold standard among instruments measuring recovery as defined by service users, the RAS-R scores were correlated with this scale in order to provide support of the construct (convergent) validity of the RAS-R.

### Ethics

All participants were regarded as able to provide consent, and all provided written informed consent. Approval for the study was sought from the Regional Committee for Medical Research Ethics (ref. no. 2009/1295). The Regional Committee for Medical Research Ethics referred the study to the Norwegian Social Science Data Services (NSD), which approved of the study (ref. no. 22920).

### Statistical analysis

A confirmatory factor analysis (CFA) was conducted to assess the relationships between the observed (items) and latent (subscales) variables according to the predefined five-factor model of the RAS-R. The extent to which the factor structure of the Norwegian sample reflected the factor structure of the original instrument in English language was assessed by comparing the covariance matrix of the sample and the corresponding matrix estimated for the population [21, 22]. Due to the non-normal sample distributions, a maximum likelihood estimation with robust standard errors (MLM) was used to compute different indices of model fit, and the Satorra–Bentler (SB) scaled χ^2^-test was used to assess the goodness of fit. The normed χ^2^, which equals the χ^2^ divided by the degrees of freedom, was reported since it is less sensitive to sample size than the χ^2^. Estimates from a maximum likelihood estimation (ML) were reported in order to allow for comparisons with other studies. The fit criteria applied were in accordance with recommendations [23–26].

Spearman’s *rho* correlation coefficient was computed between the visual analogue scale and the RAS-R total- scale. Average variance extracted (AVE) and composite reliability (CR) [27] were used to assess discriminant and convergent reliability. Convergent validity was regarded as acceptable if standardised factor loadings were > 0.50, AVE was > 0.50 and the internal consistency measure CR was larger than the AVE. Discriminant reliability was established if a factor explained more of the variance of its items than of the items belonging to other factors [28]. Further, discriminant validity was regarded as acceptable when the square root of the AVE for the factor was higher than its correlation with any other factor. As a rule of thumb, correlations between factors should be < 0.80 [24]. Factor correlations exceeding 0.80 should be scrutinised carefully from a theoretical perspective with regard to discriminant validity.

At the sub-scale level, measures of CR higher than 0.70 were considered to be a basic requirement for reliability. Further, reliability was assessed by Cronbach’s alpha, a measure of internal consistency between items within each sub-scale. Cronbach’s alphas higher than 0.70 were considered acceptable.

The lavaan package (version 0.5–16 [29]) in the R Software Package 3.0.2 (R Core team) was employed in the MLM-estimation. IBM SPSS AMOS 23 was used to assess the distribution assumptions, perform the ML-estimation, and bivariate correlations and descriptive statistics were performed using IBM SPSS 23 (Armonk, NY; IBM Corp.). Tests were two-tailed with an alpha-level of 0.05.

## Results

### Respondents

Three (1%) of the respondents were excluded due to missing responses on three or more of the RAS-items, leaving *n* = 228 with 21 or more items completed in the valid analysis file. Of these, 207 respondents completed all the RAS-R items. Missing items were replaced by the median of nearby points (span of nearby points was 2) for 21 respondents. The respondents represented a wide range of service types and mental health conditions. The sociodemographic and clinical characteristics of the respondents included in the valid analysis file (*n* = 228) are displayed in Table 2.

**Table 2:** Descriptives of the valid sample (*n* = 228)

Variable		*Mean (SD)*	Range
Age (years)		41 (14.2)	18–77
Visual Analogue Scale	In your experience, where in your process of recovery are you now, compared to where you were when things were at the worst (=1), and how you wish that your situation should ideally be (=10)	6.1 (2.04)	1–10
		Number	Percent
Gender	Man	99	43
	Woman	128	56
Highest completed level of education	Nine years compulsory school	63	28
	High school	125	55
	University/college	36	16
Civil status	Married	45	20
	Co-habiting	29	13
	Single	116	51
	Divorced/separated	23	10
	Widow (−er)	3	1.3
	Other	5	2.2
Work status^a^	Employed	43	19
	Self-employed	4	1.8
	Supported employment	3	1.3
	Domestic work	2	0.9
	Disability pension	98	43
	Student	11	5
	Out of work	7	3
	Retired	4	1.8
	Sick leave	44	19
	Other	6	2.6
Mental health service use	Out-patient, specialist services	167	63
	In-patient, specialist services	24	11
	Community mental health services	31	14
ICD-10 primary diagnosis	F00-F09 Organic, including symptomatic, mental disorders	1	0.4
	F10-F19 Mental and behavioural disorders due to psychoactive substance use	12	5
	F20-F29 Schizophrenia, schizotypal and delusional disorders	37	16
	F30-F39 Mood disorders	56	25
	F40-F48 Neurotic, stress-related and somatoform disorders	67	29
	F50-F59 Behavioural syndromes associated with physiological disturbances and physical factors	8	4
	F60-F69 Disorders of adult personality and behaviour	18	8
	F70-F79 Mental retardation	0	0
	F80-F89 Disorders of psychological development	2	0.9
	F90-F98 Behavioural and emotional disorders with onset usually occurring in childhood and adolescence	11	5
	Other	11	5

*M* means, *SD* standard deviation, *ICD-10* World Health Organization International Classification of Diseases version 10

Due to missing information frequencies and percentages do not always add up to 100

^a^Some reported more than one employment status

As for the RAS-R responses, the score distributions were explored in terms of univariate and multivariate normality. As outliers may influence parameter estimation, the Mahalanobis distance from the centroid was examined to detect potential outliers in AMOS [22]. The pre-analytic screening displayed considerable multivariate kurtosis and some outliers. Mardia’s coefficient was 157.245, and 12 observations were identified as outliers according to the Mahalanobis distance [24]. However, the outliers were perceived as true variations of the scores in the sample, as they were regarded as representative of the respondents’ experiences of their personal recoveries. Descriptive statistics of the RAS-R item responses, mean sub- and total scale scores are presented in Table 3.

**Table 3:** Descriptives of the RAS-R items and summary scales in the valid sample (*n* = 228)

Item	*Mean*	*SD*	Range	95% CI
1. I have a desire to succeed	4.6	0.62	1–5	4.56; 4.72
2. I have my own plan for how to stay or become well	3.9	0.92	1–5	3.80; 4.05
3. I have goals in life that I want to reach	4.2	0.90	1–5	4.08; 4.32
4. I believe that I can meet my current personal goals	3.7	0.98	1–5	3.59; 3.85
5. I have a purpose in life	3.8	0.94	1–5	3.72; 3.97
6. Even when I don’t care about myself, other people do	4.1	0.83	1–5	4.0; 4.21
7. Fear doesn’t stop me from living the way I want to	3.1	1.30	1–5	2.90; 3.25
8. I can handle what happens in my life	3.3	1.02	1–5	3.20; 3.47
9. I like myself	3.2	1.20	1–5	2.99; 3.31
10. If people really knew me, they would like me	3.7	0.84	1–5	3.60; 3.82
11. I have an idea of who I want to become	3.5	1.05	1–5	3.40; 3.67
12. Something good will eventually happen	4.0	0.81	1–5	3.93; 4.14
13. I’m hopeful about my future	3.9	0.99	1–5	3.72; 3.97
14. I continue to have new interests	3.7	1.09	1–5	3.57; 3.86
15. Coping with my mental illness is no longer the main focus of my life	3.1	1.23	1–5	2.98; 3.30
16. My symptoms interfere less and less with my life	3.4	1.20	1–5	3,25; 3.54
17. My symptoms seem to be a problem for shorter periods of time each time they occur	3.5	1.13	1–5	3.32; 3.61
18. I know when to ask for help	3.9	1.06	1–5	3.78; 4.05
19. I am willing to ask for help	4.1	0.92	1–5	4.01; 4.25
20. I ask for help when I need it	3.8	1.08	1–5	3.68; 3.96
21. I can handle stress	3.0	1.14	1–5	2.88; 3.18
22. I have people I can count on	4.3	0.78	1–5	4.18; 4.38
23. Even when I don’t believe in myself, other people do	4.0	0.87	1–5	3.88; 4.10
24. It is important to have a variety of friends	3.5	1.40	1–5	3.31; 3.67
Summary scales				
Personal confidence and hope (mean of items 7, 8, 9, 10, 11, 12, 13, 14 & 21)	3.5	0.68	1.67–5	3.40; 3.58
Willingness to ask for help (mean of items 18, 19 & 20)	4.0	0.89	1–5	3.84; 4.07
Goal and success orientation (mean of items 1, 2, 3, 4 & 5)	4.1	0.63	1.4–5	3.99; 4.15
Reliance on others (mean of items 6, 22, 23 & 24)	4.0	0.68	2–5	3.88; 4.06
Not dominated by symptoms (mean of items 15, 16 & 17)	3.3	0.95	1–5	3.21; 3.45
Total scale (mean of items)	3.7	0.56	1.92–5	3.66; 3.80
Total scale (sum of items)	89.5	13.39	46–120	87.75; 91.24

*M* means, *SD* standard deviation, *CI* confidence interval

### Confirmatory factor analysis

**Fig. 1:**
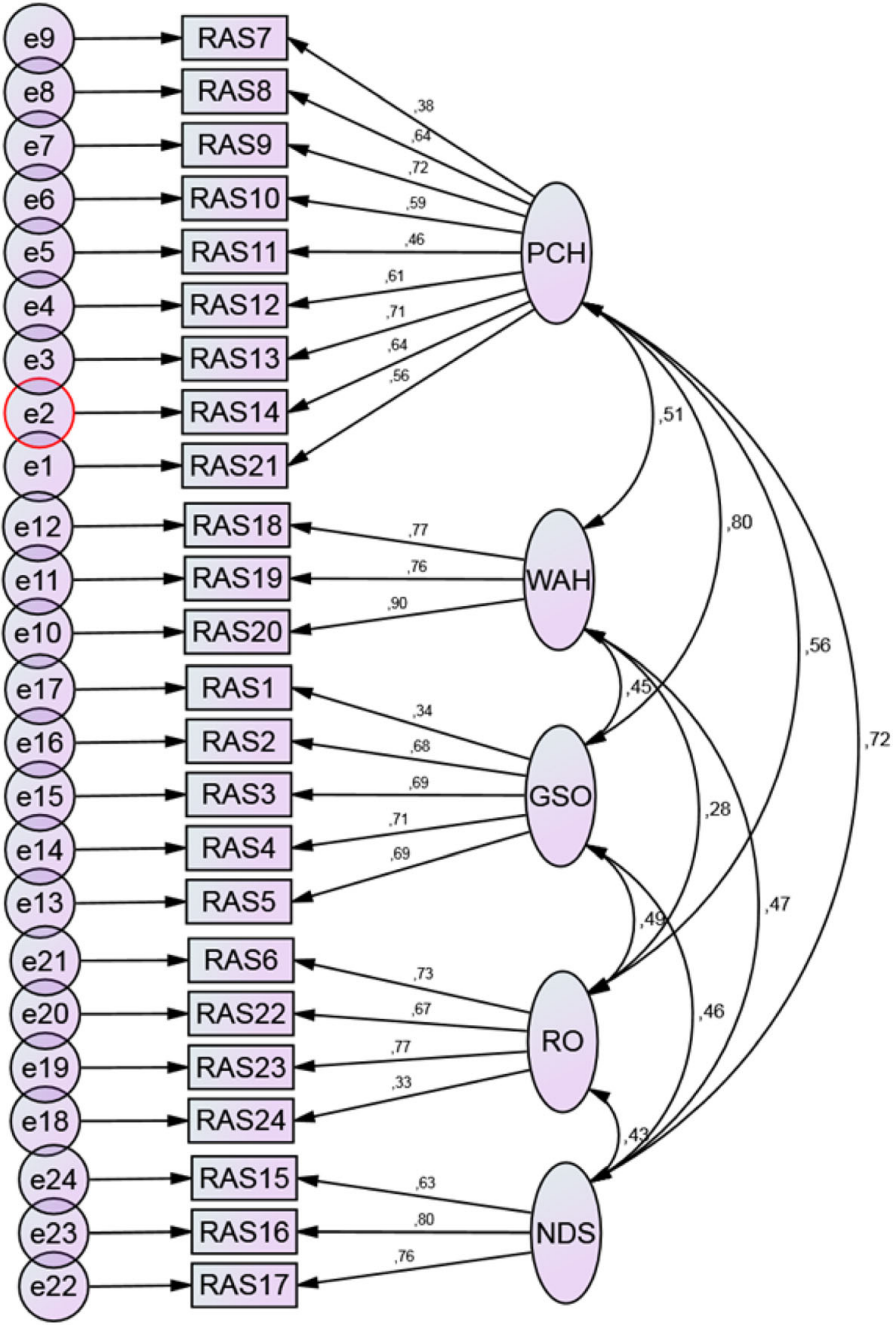
RAS-R five-factor model supplied with the standardised regression weights for each item and factor correlations. Observed variables are displayed as rectangles connected to latent factors visualised as ellipses that represent the constructs. The direction of the straight arrows indicate that observed variables should be explained by their corresponding latent factors. The two-headed curved arrows between the constructs show that they are correlated. The small circles represent measurement errors unique to each observed variable that do not contribute to explaining any variance on factor level *PCH* Personal confidence and hope (RAS 7, 8, 9, 10, 11, 12, 13, 14, 21), *WAH* Willingness to ask for help (RAS18, 19, 20), *GSO* Goal and success orientation (RAS 1, 2, 3, 4, 5), *RO* Reliance on others (RAS 6, 22, 23, 24) and *NDS* Not dominated by symptoms (RAS 15, 16, 17)

As shown in Table 4, all model fit indices, including the SRMR, which is independent of the χ^2^ [24, 30, 31], met the criteria for acceptable model fit. However, the SB χ ^2^ remained statistically significant (*p* = 0.000). The suggested model was over-identified with 300 distinct sample moments, 58 parameters and 242 degrees of freedom. Figure 1 shows the resultant five-factor model with the standardised regression weights for each item and factor correlations. Table 5 shows the parameter estimates with standard errors (SE) from the CFA.

**Table 4:** Fit criteria and global fit assessed by robust Satorra–Bentler (MLM) and ML estimators (*n* = 228)

Model	Fit-criterion	MLM-robust	ML
χ^2^		360.699	493.608
*p*	> .05	.000	.000
*df*		242	242
Normed χ^2^	< 5.0	1.49	2.04
CFI	≥ .90	0.924	0.876
TLI	≥ .90	0.913	0.858
RMSEA	≤ .06	0.046	0.068
RMSEA 90% CI	.06–.08	.038–.055	.059–0.076
SRMR	≤ .08	0.063	0.063

*ML* maximum likelihood, *MLM* maximum likelihood mean-adjusted, *df* degrees of freedom, *CFI* Bentler comparative fit index, *TLI* Tucker–Lewis incremental index, *RMSEA* Steiger–Lind root mean square error of approximation, *CI* confidence interval, *SRMR* standardised root mean square residual

**Table 5:** Standardised (β) factor loadings, communalities, and unstandardised (B) factor loadings with standard errors (SE)

Personal confidence and hope-factor				
Item	β	Communality	B	SE
RAS7	0.38	0.15	0.79	0.16
RAS8	0.64	0.41	1.04	0.14
RAS9	0.72	0.51	1.38	0.17
RAS10	0.59	0.35	0.78	0.11
RAS11	0.46	0.21	0.77	0.13
RAS12	0.61	0.37	0.78	0.11
RAS13	0.71	0.50	1.11	0.14
RAS14	0.64	0.41	1.11	0.15
RAS21	0.56	0.31	1.00	
Willingness to ask for help-factor				
Item	β	Communality	B	SE
RAS18	0.77	0.59	0.83	0.07
RAS19	0.76	0.68	0.72	0.06
RAS20	0.90	0.81	1.00	
Goal and success orientation-factor				
Item	β	Communality	B	SE
RAS1	0.35	0.13	0.33	0.07
RAS2	0.68	0.46	0.97	0.11
RAS3	0.69	0.48	0.96	0.11
RAS4	0.71	0.50	1.07	0.12
RAS5	0.69	0.48	1.00	
Reliance on others-factor				
Item	β	Communality	B	SE
RAS6	0.73	0.53	1.35	0.30
RAS22	0.68	0.46	1.16	0.27
RAS23	0.77	0.59	1.48	0.33
RAS24	0.33	0.11	1.00	
Not dominated by symptoms-factor				
Item	β	Communality	B	SE
RAS15	0.63	0.40	0.90	0.11
RAS16	0.80	0.64	1.06	0.10
RAS17	0.76	0.58	1.00	

### Convergent and discriminant validity

Table 6 provides vital information for assessing convergent and discriminant validity issues and shows correlations between items, between items and factors and inter-factor correlations. Convergent validity was supported, as all the items loaded mostly on their respective parent factors. Further, the findings that most standardised factor loadings (range 0.33–0.90) (Table 5) and correlations between items within each factor (range 0.47–0.90) (Table 6) were moderate to high supported convergent validity, implying that the latent factors were explained by their items. However, four of the twenty-nine factor loadings (RAS1 (standardised factor loading 0.35), RAS7 (0.38), RAS11 (0.46), and RAS24 (0.33)) were below the basic requirement of 0.5 for establishing convergent validity at the item level (Table 5). The items RAS1, RAS7 and RAS24 had the lowest correlations with their parent factors (0.34, 0.38 and 0.33, respectively). Comparing the CR values to the AVE values across factors revealed that all CRs were higher than their respective AVEs, indicating good convergent validity. Convergent validity for the factors Willingness to ask for help and Not dominated by symptoms were further supported by AVE values > 0.50. However, AVE estimates for the other three factors were below the threshold of 0.50 for convergent validity at the sub-scale level (Table 7).

**Table 6:** Correlations between items, items and factors and inter-factor correlations

	NDS	RO	GSO	WAH	PCH	RAS15	RAS16	RAS17	RAS6	RAS22	RAS23	RAS24	RAS1	RAS2	RAS3	RAS4	RAS5	RAS18	RAS19	RAS20	RAS7	RAS8	RAS9	RAS10	RAS11	RAS12	RAS13	RAS14	RAS21
NDS	1.00																												
RO	0.43	1.00																											
GSO	0.46	0.49	1.00																										
WAH	0.47	0.28	0.45	1.00																									
PCH	^a^0.72	0.56	^a^0.80	0.51	1.00																								
RAS15	0.63	0.27	0.29	0.29	0.45	1.00																							
RAS16	^a^0.80	0.35	0.37	0.37	0.58	0.50	1.00																						
RAS17	^a^0.75	0.32	0.35	0.35	0.55	0.47	0.61	1.00																					
RAS6	0.31	^a^0.73	0.36	0.20	0.41	0.20	0.25	0.24	1.00																				
RAS22	0.29	0.68	0.33	0.19	0.38	0.18	0.23	0.22	0.49	1.00																			
RAS23	0.33	^a^0.77	0.38	0.21	0.43	0.21	0.27	0.25	0.56	0.52	1.00																		
RAS24	0.14	0.33	0.17	0.09	0.19	0.09	0.12	0.11	0.24	0.23	0.26	1.00																	
RAS1	0.16	0.17	0.35	0.15	0.28	0.10	0.13	0.12	0.12	0.12	0.13	0.06	1.00																
RAS2	0.31	0.33	0.68	0.30	0.54	0.19	0.25	0.23	0.24	0.23	0.26	0.11	0.23	1.00															
RAS3	0.32	0.34	0.69	0.31	0.55	0.20	0.25	0.24	0.25	0.23	0.26	0.11	0.24	0.47	1.00														
RAS4	0.32	0.35	^a^0.71	0.32	0.56	0.20	0.26	0.25	0.26	0.24	0.27	0.12	0.24	0.48	0.49	1.00													
RAS5	0.32	0.34	0.69	0.31	0.55	0.20	0.25	0.24	0.25	0.23	0.26	0.11	0.24	0.47	0.47	0.49	1.00												
RAS18	0.36	0.21	0.34	^a^0.77	0.39	0.22	0.29	0.27	0.16	0.14	0.16	0.07	0.12	0.23	0.24	0.24	0.24	1.00											
RAS19	0.35	0.21	0.34	^a^0.76	0.39	0.22	0.28	0.27	0.15	0.14	0.16	0.07	0.12	0.23	0.23	0.24	0.23	0.58	1.00										
RAS20	0.42	0.25	0.40	^a^0.90	0.46	0.26	0.34	0.32	0.18	0.17	0.19	0.08	0.14	0.27	0.28	0.29	0.28	0.69	0.68	1.00									
RAS7	0.28	0.22	0.31	0.20	0.38	0.17	0.22	0.21	0.16	0.15	0.17	0.07	0.11	0.21	0.21	0.22	0.21	0.15	0.15	0.18	1.00								
RAS8	0.47	0.36	0.51	0.33	0.64	0.29	0.37	0.35	0.26	0.24	0.28	0.12	0.18	0.35	0.35	0.36	0.35	0.25	0.25	0.30	0.25	1.00							
RAS9	0.52	0.40	0.57	0.37	^a^0.72	0.32	0.42	0.39	0.29	0.27	0.31	0.13	0.20	0.39	0.39	0.41	0.39	0.28	0.28	0.33	0.28	0.46	1.00						
RAS10	0.42	0.33	0.47	0.30	0.59	0.27	0.34	0.32	0.24	0.22	0.25	0.11	0.16	0.32	0.32	0.33	0.32	0.23	0.23	0.27	0.23	0.38	0.42	1.00					
RAS11	0.33	0.26	0.37	0.24	0.46	0.21	0.27	0.25	0.19	0.17	0.20	0.09	0.13	0.25	0.25	0.26	0.25	0.18	0.18	0.21	0.18	0.30	0.33	0.27	1.00				
RAS12	0.44	0.34	0.49	0.31	0.61	0.28	0.35	0.33	0.25	0.23	0.26	0.11	0.17	0.33	0.34	0.35	0.34	0.24	0.24	0.28	0.23	0.39	0.44	0.36	0.28	1.00			
RAS13	0.51	0.40	0.57	0.36	^a^0.71	0.32	0.41	0.39	0.29	0.27	0.31	0.13	0.20	0.38	0.39	0.40	0.39	0.28	0.27	0.33	0.27	0.46	0.51	0.42	0.33	0.43	1.00		
RAS14	0.46	0.36	0.51	0.33	0.64	0.29	0.37	0.35	0.26	0.24	0.28	0.12	0.18	0.35	0.35	0.36	0.35	0.25	0.25	0.29	0.25	0.41	0.46	0.38	0.30	0.39	0.46	1.00	
RAS21	0.40	0.31	0.44	0.28	0.56	0.25	0.32	0.30	0.23	0.21	0.24	0.10	0.15	0.3	0.31	0.31	0.31	0.22	0.22	0.26	0.21	0.36	0.40	0.33	0.26	0.34	0.40	0.36	1.00

^a^Correlation coefficients ≥0.70

**Table 7:** Convergent and discriminant validity test with factor correlations

	CR	AVE^a^	Personal Confidence and Hope	Willingness to Ask for Help	Goal and Success Orientation	Reliance on Others	Not Dominated by Symptoms
Personal confidence and hope	0.830	0.359	*0.599*	0.429			
Willingness to ask for help	0.851	0.657	0.429	0.811			
Goal and success orientation	0.764	0.401	0.611	0.434	*0.633*		
Reliance on others	0.732	0.423	0.462	0.286	0.407	0.650	
Not dominated by symptoms	0.774	0.536	0.620	0.423	0.354	0.362	0.732

*CR* composite reliability, *AVE* average variance extracted (squared factor loadings averaged)

Diagonals show square roots of AVE; values in *italics* may indicate discriminant validity issues (i.e. factors other than the parent factor may explain more of the item variance)

^a^Acceptable levels for AVE > 0.5 and CR ≥ AVE

The bivariate correlation (Spearman’s *rho*) was 0.59 (*p* < 0.000) between the participants’ responses to the question ‘In your experience, where in your process of recovery are you now, compared to where you were when things were at the worst (=1), and how you wish that your situation should ideally be (=10)’ and the RAS-R total scale.

Discriminant validity was supported by the finding that each parent factor explained more of the variance of its items than of the items belonging to the other factors. Comparing the square roots of the AVEs of the factors (the diagonals in Table 7) to their respective factor’s correlation with any other factor (Table 6) revealed that the discriminant abilities of three factors were acceptable. However, the discriminant abilities of the factors Goal and success orientation and Personal confidence and hope could be questioned. The factor Goal and success orientation factor showed a factor correlation at the limit of 0.80 for discriminant validity (Table 6). The inter-construct correlations of the Goal and success orientation factor with the Personal confidence and cope-factor, and the Personal confidence and hope factor with the Not dominated by symptoms factor, were higher than the square roots of their respective AVEs (Table 7). Some correlation, or redundancy, was present between factors (i.e. correlation coefficients ≥0.70; Table 6).

### Reliability

Indicator reliability, as measured by the explained variance in Table 5, ranged from a lower communality, 0.11 for RAS24, to the higher end, 0.81 for RAS20. At the sub-scale level, all CRs were well above 0.70 (Table 7). Further, internal consistencies within sub-scales as measured by Cronbach’s alpha were 0.83 for the Personal confidence and hope, 0.85 for the Willingness to ask for help, 0.77 for the Goal and success orientation, 0.65 for the Reliance on others and 0.76 for the Not dominated by symptoms factors, respectively. Cronbach’s alpha for the total scale was 0.90.

## Discussion

The present study supports the Norwegian version of the RAS-R for use in the Norwegian language and context. The translation of the instrument into the Norwegian language was thorough and included feedback from service users with long experience of severe mental health issues. The pilot study indicated that the instrument was acceptable and feasible for service users. A few comments made by the respondents during the piloting suggested challenges with the comprehensibility of item 5 (‘I have a purpose in life’) and items that were negatively worded. These challenges were, however, minor and did not lead to any re-phrasings that would make the Norwegian version of the RAS-R deviate from the original U.S. English version.

Earlier studies have concluded that the core values and concepts of recovery have transcultural relevance [32–34]. Mean RAS-R scores in the present study were comparable to mean scores in earlier studies using the RAS or RAS-R, most of which were performed in the English-speaking part of the world [13]. However, a transcultural study performed in Japan and the US found differences in RAS-R response patterns between the two countries [34]. The lower means on the Goal and success orientation and Reliance on others sub-scales reported by Japanese respondents, as compared to US respondents, was explained by differences in emphasis of aspects of hope, personal confidence and collectivism between Eastern and Western cultures. In the present study mean RAS-R scores appeared similar to the scores reported by Cavelti et al. (2017) in their recent study from the German cultural context [35]. The fact that mean RAS-R responses in the Norwegian context were similar to the responses in the study set in the German context, may reflect similarity in cultures among these two countries. For instance, the way psychological constructs such as personal recovery are viewed, and the interpretation and use of language, may be similar. Probably, the similarity in mean RAS-R scores found in the present study compared to earlier studies in the Western world [13, 35] also indicates high validity of the newly translated Norwegian version of the RAS-R. Therefore results from studies using the Norwegian RAS-R may be transferred to countries belonging to the same cultural sphere.

In the present study the results from the CFA, i.e. the goodness-of-fit estimates and parameter estimates, yielded acceptable results, supporting the established five-factor structure from the original U.S. RAS-R version [18]. The construct (convergent) validity of the RAS-R was supported by the correlation between the RAS-R and the visual analogue scale representing responses to the question ‘In your experience, where in your process of recovery are you now, compared to where you were when things were at the worst (=1), and how you wish that your situation should ideally be (=10)’. This visual analogue scale was used to operationalise the construct of ‘personal recovery’ in the present study, as no ‘gold-standard’ measure of this construct exists. The validity of the visual analogue scale was probably lower than any ‘gold-standard’ measure of personal recovery would have been. However, although the correlation between the RAS-R and the visual analogue scale probably was weakened due to lower validity of the visual analogue scale, we argue that the correlation found between RAS-R and the visual analogue scale in the present study supports the convergent validity of the Norwegian RAS-R. Convergent validity was further supported since all the items loaded mostly on their respective parent factors, most standardised factor loadings and correlations between items within each factor were moderate to high and the CRs were above the AVEs of the respective factors. This means that the latent factors (sub-scales) were supported by their items. However, convergent validity was not completely supported for the Personal confidence and hope, Goal and success orientation and Reliance on others factors, as their AVE values were below the levels usually regarded as acceptable. Reliability and internal consistency of the RAS-R sub-scales were found to be good. The discriminant ability of the Personal confidence and hope factor may, however, be questionable, as the square root of the AVE of this factor was slightly larger than the factor’s correlation with the Goal and success orientation and Not dominated by symptoms factors, suggesting some overlap between these sub-scales. However, some degree of overlap between sub-scales may be expected within instruments measuring psychosocial constructs. In the present study, the inclusion of service users with a wide range of types of mental health problems probably reduced the risk of sampling biases and increased the reliability and generalisability of the findings. As personal recovery is a longitudinal process that occurs in stages [4, 20], participants who had experienced partly or total recovery was included in order to increase variance in the construct explored. However, some limitations need to be discussed. One concern is the fact that the back-translation of the Norwegian RAS-R into English language was evaluated by the research team and not by independent experts. However, inconsistencies between the back-translated version and the original version in U.S. English were very minor and thus it is unlikely that this limitation lead to inconsistency between the original and the translated version. Although visual inspection revealed no systematic pattern in missing item responses, we cannot exclude the possibility that replacing missing items lead to biases in responses. Another concern is the rejection of the predefined measurement model by the SB χ^2^ test. Sample size, communalities and model complexity influence the SB χ^2^ test, which is not considered reliable for *N* > 200 samples [24, 31, 36]. However, in the present study, the normed χ^2^ was within acceptable ranges [23]. Consequently, the conclusion about how well the proposed model fitted was based on both the theory and the evaluation of the indices of model fit, the normed χ^2^ and relevant estimates of validity and reliability, rather than the SB χ^2^-test. Finally, the data were cross-sectional and did not allow for assessment of instrument properties such as test–retest reliability and responsiveness to change.

## Conclusions

We conclude that the RAS-R is an acceptable, feasible, valid and reliable tool for assessing mental health recovery, as defined and experienced by service users, in the Norwegian language and context. Hopefully, the Norwegian RAS-R will become a useful tool for service users and health professionals in their collaborative work towards the service users’ recovery goals. Patient-based outcome measures such as the RAS-R should be used in future evaluations and research in order to increase the validity of findings by capturing central features of mental health recovery that are not captured by traditional clinical instruments. Future studies assessing the psychometric properties of the RAS-R should have longitudinal designs in order to allow for the evaluation of test–retest reliability and responsiveness.

## Abbreviations

AVE: Average variance extracted
CFA: Confirmatory factor analysis
CFI: Bentler comparative fit index
CI: Confidence interval
CMHC: Community Mental Health Centre
CR: Composite reliability
Df: Degrees of freedom
GSO: Goal and success orientation sub-scale
ML: Maximum likelihood estimation
MLM: Maximum likelihood estimation with robust standard errors
NDS: Not dominated by symptoms sub-scale
PCH: Personal confidence and hope sub-scale
RAS-R: Recovery Assessment Scale-revised
RMSEA: Steiger–Lind root means square error of approximation
RO: Reliance on others sub-scale
SB: Satorra–Bentler
SD: Standard deviation
SE: Standard error
SRMR: Standardised root mean square residual
TLI: Tucker-Lewis incremental index
WAH: Willingness to ask for help sub-scale
